# Supplementary material for: Candidate Causal Variants at the 8p12 Breast Cancer Risk Locus Regulate DUSP4
Source: Cancers (Basel). 2020 Jan 10;12(1):170. doi: 10.3390/cancers12010170 (PMC7016765; doi:10.3390/cancers12010170)
Supplement: Supplementary file 1 [file cancers-12-00170-s001.zip › Supp Fig 1_luciferase assay_revised.pdf]

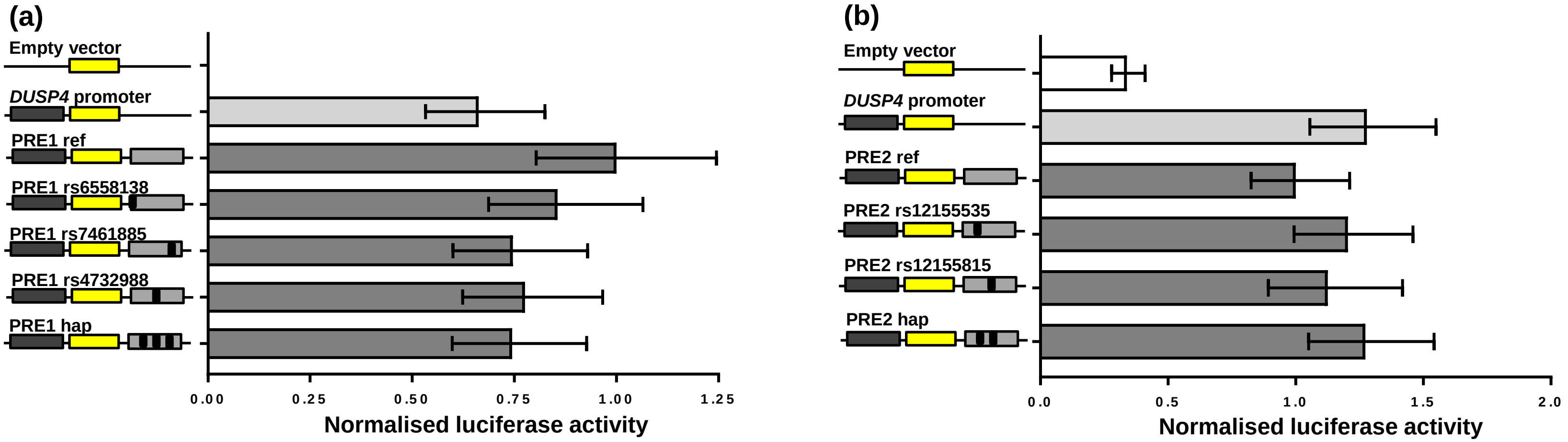

Supplementary Figure S1. Luciferase reporter gene analysis of PRE1 and PRE2 in T-47D cells. PRE1 and PRE2 reference regions, containing protective allelic variants of CCVs, were cloned downstream of a *DUSP4* promoter luciferase construct for the creation of reference (ref) constructs. Risk allelic variants of CCVs were engineered into the constructs and are designated by the rs ID of the corresponding variant. Constructs containing risk allele haplotypes (hap) were also generated. Cells were transiently transfected with each of these constructs and assayed for luciferase activity after 24 h. Panels show back-transformed data for: **(a)** PRE1 activity and **(b)** PRE2 activity. Error bars denote 95% confidence intervals of experiments performed in triplicate. P-values were determined two-way ANOVA followed by Dunnett's multiple comparisons test.
